# Commencement in the University of Louisville

**Published:** 1849-04

**Authors:** 


### COMMENCEMENT IN THE UNIVERSITY OF LOUISVILLE.

The degree of M.D was conferred on eighty-one of the alumni of the University of Louisville on the evening of the 5th of March. The Hon. James Guthrie, President of the Board of Trustees, tendered to the graduates the congratulations of the Board, and urged upon them, in impressive terms, the duty of diligence in professional study as the only course by which they could hope to reflect credit upon the institution, the highest honors of which they were now about bearing to their distant homes. The valedictory address on the part of the Faculty was delivered by Professor Caldwell, and occupied in its delivery about two hours. It took a wide range. The present state of the medical profession as compared with its condition in former years, in the United States and in Europe; the physical grandeur of the American continent, our mountain ranges, rivers, lakes, and prairies; the superiority of the Anglo-Saxon race, and its destiny; our political institutions; physical education, and sectional medicine, all were discussed in the characteristic style of the venerable professor.

The number of students in the medical department of the University of Louisville during the last session was 331. The Annual Catalogue and Circular will soon be issued.

